# Goal choice in preschoolers is influenced by context, cognitive flexibility, and metacognition

**DOI:** 10.3389/fpsyg.2022.1063566

**Published:** 2023-02-23

**Authors:** Marion Leclercq, Guillaume Gimenes, Célia Maintenant, Jérôme Clerc

**Affiliations:** ^1^Univ. Lille, ULR 4072 – PSITEC – Psychologie: Interactions, Temps, Emotions, Cognition, Lille, France; ^2^Laboratory PAVeA, Department of Psychology, University of Tours, Tours, France; ^3^Laboratory CNRS LPNC, Department Psychology, University of Grenoble Alpes, Grenoble, France

**Keywords:** goal choice, self-regulated learning, cognitive flexibility, metacognition, early childhood

## Abstract

**Introduction:**

Goal choice is one of the first, and most important, steps in self-regulated learning (SRL). It is particularly challenging for young children (before 5–6 years), who tend to rely on available environmental cues, which makes their goals fragile because of the instability and variability of the environment. Therefore, it can be assumed that the conditions under which a task is performed may influence a child’s learning goal choice. Moreover, adapting to constraints involves control capacities provided by executive functions (EF) and metacognition.

**Methods:**

The main purpose of this study was to determine factors that influence the way preschoolers choose a learning goal during the first step of SRL. We tested whether adding constraints to perform a task may influence the choice of the procedure that a child aims to learn to perform this task. We also examined the role of cognitive flexibility and metacognition in goal selection in the face of these changes, and tested the influence of change over time, comparing participants’ performance at two points in the school year. One hundred 4-year-olds were asked to perform a jigsaw puzzle task under two conditions: predictable vs. unpredictable environmental change. Individual levels of cognitive flexibility and metacognition were also measured.

**Results:**

The results show that only a predictable change, but not an unpredictable one, leaded children to change their learning goals. Furthermore, when participants were faced with an unpredictable change, metacognition and cognitive flexibility significantly predicted learning goal change. Results are discussed regarding the development of SRL, flexibility, and metacognition. Educational suggestions are proposed.

**Highlights**

– The choice of a learning goal by a preschooler is influenced by the conditions of task performance and environmental cues. Facing a predictable change is more disruptive to children before the age of 4.5, and more likely to cause them to change their goal.
– A shift is observed, from age 4 and during the school year, from a perceptual to a conceptual level of processing.
– Cognitive flexibility and metacognition also determine learning goal choice in preschoolers, but only in front of unpredictable changes.

## 1. Introduction

Self-regulated learning (SRL) consists of the specific processes by which a learner mobilizes knowledge and strategies according to the context, in order to organize and control its learning (Zimmerman, 1990). It underpins the learner’s abilities to reflect, adapt, and adjust when faced with changing conditions in the environment, thus linking personal and contextual characteristics in a learning situation. In these specific processes, three phases can be identified in SRL (Zimmerman, 2013). The first one (*“Forethought Phase”*) is a preparation phase, where learners analyze the task and build a strategic plan to achieve the goal. Before engaging in the activity, they identify the resources available to them, in order to choose learning goals and draft an appropriate strategic plan. The second phase (*“Performance Phase”*) is a commitment and monitoring phase occurring during the activity. Learners execute the strategic plan, monitor the progress of their work (bottom-up process) and adapt their strategies if necessary (top-down process). The third phase (*“Self-Reflection Phase”*) is an evaluation and reflection phase, once the activity has been completed. Learners evaluate the learning process that has just taken place and decide to keep their strategies during subsequent learning or to modify them. One of the major issues in SRL is how effectively learners will be able to select, combine and coordinate the strategies available to them in their repertoire of effective strategies for learning (Boekaerts, 1999; Winne, 2011, 2018). A cognitive strategy is considered as an intentional and deliberate processing of information that will improve one’s performance (Bjorklund et al., 1992). However, although goals and strategies are linked, in this study, we focused exclusively on what happens before, during the first stage of the SRL (*“Forethought Phase”*), namely choosing the goal and determining more precisely which factors could influence this decisive stage in preschoolers. The Forethought phase is indeed of particular interest as it is the starting point of the SRL, and no learning would be possible without this first step. It consists of preparing to enter into the action by activating one’s representations, knowledge, and motivational beliefs concerning the task and its context (Boekaerts, 1999). It includes identifying and choosing a goal, as well as planning how to achieve it by using the appropriate knowledge and strategies (Winne, 2011, 2018; Zimmerman, 2013). Goal choice is at the heart of SRL, especially in its first step which is the Forethought Phase.

Goals are a determining factor in the decision-making process. A goal can be seen as “an internal representation of a desired states where states are broadly construed as outcomes, events, or processes” (Austin and Vancouver, 1996, p. 338). According to Schunk (1990), a goal is “what an individual consciously tries to accomplish” (p. 71). Thus, a goal is both the outcome to be achieved and what guides action by giving it direction and energy. Goals have several functions, such as eliminating unrealistic options or indicating what information should be gathered and what plans should be developed to achieve them (Galotti, 2005). Defining a goal requires an accurate analysis of the task to be performed, which occurs during the Forethought Phase of Zimmerman (2013) SRL model. We argue that a distinction must be made. We can then oppose between two main types of goals, the “practicing” goals and the learning goals. A practicing goal consists of knowledge or skill already mastered, that the student can immediately apply to the task. A learning goal is knowledge or skill that the student aims to learn through the task. Following the SRL framework, a learning goal refers to the specific knowledge, skill or procedure that the child does not yet have, and that it aims to acquire through the task, as it is typically the case at school. Furthermore, a learning goal choice is mandatory in any learning task, that is any task intended to make children acquire new knowledge or skills. Choosing a learning goal requires both a correct understanding of the task and its constraints; and an evaluation of its difficulty, in relation to the environmental changes. Lastly, this is a dynamic process since modifications constrained by a task may lead to change one’s goal. In this sense, goal choice is possible thanks to contextual cues, including information about the task’s context and the use of learning strategies that signal the need for change (Boekaerts et al., 2000; Pino-Pasternak et al., 2014; De Bruin and Van Merriënboer, 2017; Roebers et al., 2019).

Choosing a goal is very hard for young children, and they still need guidance and support to achieve it (Lucenet and Blaye, 2014, 2019; Doebel et al., 2018; Perry, 2019; Roebers et al., 2019). In complex tasks, young children often struggle to analyze the task to be performed and the goal that they must pursue. In the absence of any explicit indication, and especially when faced with a new task, must often they deduce the goal of the task from available environmental cues. They do this by activating their limited conceptual knowledge and evaluating the task’s difficulty (Chevalier et al., 2014; Blaye, 2021). Young children’s cognitive abilities, necessary to account for such cues, are still developing though, which prevents them from being fully aware of all aspects of a learning task. This is the case in many school situations when faced with new learning. Indeed, choosing a learning goal does not guarantee that it will be reached on the first try, because the child does not know yet how to solve the task or the problem. Thus, often, the learning objectives targeted by teachers are more about the procedures to be mastered by the child than about the result itself (for example, in mathematics, aiming more at the operating technique than at the final result). Furthermore, choosing a learning goal is not enough to achieve it, as certain contextual elements can distract a child from learning (Chevalier, 2015). A learning goal can thus encourage individuals to use their knowledge and abilities in the task, but individuals must also be able to adapt their behavior, especially when faced with new complex tasks or with environmental changes. Moreover, this ability to adapt to a learning task, which sometimes results in the child modifying its goal, is fueled by cognitive control capacities.

As children grow older, they tend to better regulate their behaviors in complex situations, thanks to improvements in cognitive control (Diamond, 2013; Thaqi and Roebers, 2020). This improvement is often interpreted as a shift in the mode of cognitive control that occurs during preschool. Cognitive control gradually evolves from a reactive, cue-dependent and contextually influenced mode, to a more proactive and anticipatory mode which is less dependent on the context (Braver, 2012; Munakata et al., 2012; Chevalier et al., 2017). Recent research shows that children aged 4–6 cannot yet use proactive control spontaneously (Gonthier and Blaye, 2022). The shift from reactive to proactive control, which is observed around the age of 6, reflects progress in SRL abilities, including goal choice (Blackwell and Munakata, 2014; Zelazo, 2015; Gonthier et al., 2019). Furthermore, the progression towards proactive control is linked to better, more precise goal representation (Chevalier and Blaye, 2016). Thus, when choosing a goal in a practicing task, children are likely to depend less and less on the environment as they grow up, benefitting from more and more proactive cognitive control. Indeed, younger children seem more easily distracted than older children when a change occurs in the task (Blakey et al., 2016). We argue that it may be the case in learning tasks too. Furthermore, proactive control includes several dimensions among which cognitive flexibility plays an important role.

Cognitive flexibility is the ability to adapt one’s thoughts and behaviors in response to changes in our goal or environment (Blakey et al., 2016). With working memory updating and inhibition, it is one of the abilities comprised in the executive functions (EF), a set of control processes necessary to carry out goal-directed behavior (Diamond, 2016). EF helps controlling one’s cognitive processes in a targeted and context sensitive manner, in new or unexpected situations (Miyake et al., 2000; Miyake and Friedman, 2012). Cognitive flexibility, specifically, allows us to reconfigure our mind quickly to be able to switch between tasks or strategies (Braem and Egner, 2018). During the execution of an activity, it allows a child to analyze the environment from different angles. It also allows us to adapt, by conceiving new ways of considering the situation or by developing new procedures or new goals. Two sides of flexibility can be distinguished: one attentional and the other conceptual (Chevalier et al., 2014; Maintenant and Pennequin, 2016). Indeed, flexibility corresponds to a set of processes related to task shifting (attentional part), as well as decision making and goal choosing (conceptual part). Interestingly, these two sides of flexibility have never been studied together in young children, which is damageable since executive function develop dramatically between the ages of 2 and 6. In practicing tasks, flexibility seems to be involved in goal choice, since a task’s goal is constantly re-evaluated and sometimes modified. Faced with an unstable environment, cognitive flexibility allows for quickly changing strategies or goals during the task (Cragg and Chevalier, 2012). It thus underlies an individual’s ability to choosing goals and achieving them (Blaye and Chevalier, 2011; Hendry et al., 2016). A lack of flexibility may cause difficulties in identifying the task’s goal when it is based on environmental cues, which in turn may prevent a child from choosing a clearly defined personal goal. We hypothesized that it may be the case also in learning tasks. Furthermore, flexibility is not the only function involved in goal change, as metacognition could just as easily be involved.

Metacognition refers to knowledge, monitoring and control of one’s cognitive processes to reach a goal (Flavell, 1979). It is a multifaceted concept, and two aspects can be especially distinguished, metacognitive knowledge and metacognitive skills (Flavell, 1976; Efklides, 2011). Metacognitive knowledge is cognition about cognition that individuals consciously build up about their mental acts as they experience them. It includes self-awareness, and it is enabled by a knowledge base containing information about the conditions for using various cognitive strategies. Metacognitive skills are a set of various strategies which ensure the control over one’s cognition and which can be used in any cognitive task. They are composed of mechanisms of control and monitoring (Nelson and Narens, 1990) allowing for self-regulation that promote access and production of cognitive strategies. Thus, metacognition takes on different aspects in learning (Efklides, 2008): what can be learned (*about what?*), when can it be learned (*when?*), according to the metacognitive skills involved (*according to what?*), according to the tools that can be used (*how?*). When faced with a cognitive task, children develop their metacognitive processes, which allow them to reflect on their actions and their consequences. They can also decide to modify their actions (Efklides, 2011, 2014), especially if their goal changed during the course of the task. Indeed, metacognition can also be involved in choosing or changing goals during a task, since this requires mobilizing metacognitive knowledge and metacognitive skills. Goals are a set of norms that learners use to metacognitively track their progress in the task and modify their actions to adjust to the task’s demands (Efklides, 2011, 2014; Winne, 2018). Hence, by comparing their knowledge and their application of it when performing a task, they can adjust their behavior by modifying what they do next: in other words, show metacognitive control (Winne, 2011). Metacognition can therefore be involved in changing one’s learning goal during a task.

Recent work points to need for multiple, more ecological tools and procedures that would capture this ability to adapt to change (Diamond, 2016; Follmer and Sperling, 2016; Marulis et al., 2016, 2020; Moreno et al., 2017; Roebers, 2017; Zachariou and Whitebread, 2019). Thus, we looked for a task that would be particularly appropriate for young children and chose to use a jigsaw puzzle task. This game is familiar to young children and easily found in preschool classes. It has many interests, both in terms of motor skills (hand-eye coordination, fine motor skills, and spatial skills) and reasoning skills by supporting the use of problem-solving strategies and specific mathematical skills (Aral et al., 2012; Levine et al., 2012; Doherty et al., 2021). Moreover, it essentially calls upon visual–spatial cognitive processes, which limits the use of language processes, known to give rise to significant inter-individual differences in young children. On the other hand, completing a jigsaw is likely to mobilize cognitive flexibility, as studies have shown that flexibility is involved in spatial representation and drawing (Picard and Vinter, 2005; Ebersbach and Hagedorn, 2011). Metacognition is also likely to be involved. Indeed, while constructing a jigsaw puzzle, a child can check the correspondence with the completed image (monitoring) to make adjustments in the construction and assembly of the pieces (control; Marulis et al., 2020). The first signs of metacognitive control have been found around 3–4 years of age in play situations (Destan et al., 2014; Roebers and Spiess, 2017), especially in a jigsaw task (Sperling et al., 2000). Finally, completing a jigsaw involves understanding that a picture will be produced once the various pieces are assembled and combined, thus linking this activity to metarepresentational development (Doherty et al., 2021).

A jigsaw puzzle admits only one goal, which is to build it by assembling all the pieces together. However, this necessitates *knowing how* to put all the pieces together correctly. This is not easy for young children who may use different procedures to realize this complex activity. Indeed, not all children engage in the jigsaw in the same way. For example, Levine et al. (2012) showed that few young children-i.e., 2–4 years old-spontaneously engage in this type of play when confronted with it. Observing child–parent dyads in play interactions, they found that barely half of their sample (27/53 dyads) did so. They also showed that jigsaw completion is mainly influenced by experience (frequency of use and quality of play) as well as spatial transformation skills. Furthermore, the ability to complete jigsaw puzzles improves significantly between the ages of 3 and 5 years. At ages 4 and 5 in particular, children rely primarily on available cues of shape or pictorial content (Doherty et al., 2021). Thus, in preschool, we can observe a great variability in the procedures for completing a jigsaw, partially depending on familiarity with the task. At age 4, most children have not yet learned a stable and permanent way to complete a jigsaw. They understand that the goal is to arrange all the pieces together, but they do not yet know how to do it. So, they need to learn what procedures might allow them to do so. Facing a jigsaw puzzle is thus a learning situation in which a young child should learn the procedure for assembling the pieces. This procedure thus constitutes a learning goal typical of the Forethought Phase of SRL. Moreover, no formal teaching of these procedures takes place in preschool, at least in France.

To summarize, whether facing complex tasks or learning new knowledge, procedure or skills, young children often rely on environmental cues to choose the goal to be pursued. Consequently, since practicing tasks are likely to be influenced by environmental constraints (Blakey et al., 2016), it can fragilize this choice. For the same reason, we hypothesize that by manipulating the environment, it could modify the goals and plans of young children even if the changes are not appropriate. It is not uncommon to see this phenomenon in preschools classes. Distracted by the context, children quickly lose sight of the original goal of the task at hand by adopting sometimes surprising behaviors.

The current study aims to better understand the factors that influence SRL processes, such as the task and/or contextual characteristics (Cleary et al., 2012; Gorges and Göke, 2015; Depover et al., 2016), as well as the factors that determine its development in young children (Jacob et al., 2019a,b; Dörr and Perels, 2020; Marulis et al., 2020). The period between 3 and 5 years of age is key for the development of SRL, EF, and metacognitive skills. In addition, by age 4, most children know that the goal of a jigsaw puzzle is to put all the pieces together, but they do not yet know how to do this using a stable and permanent procedure. Specifically, we considered the specific procedure that the child intended to use, among the different procedures available to him for completing the jigsaw, as the learning goal. We hypothesized that the presence of an environmental constraint in a jigsaw task would promote a change in the child’s goal, that is a change in the specific procedure that he/she aims to learn and apply to the task. Thus, children should tend to change their goal in constrained conditions. The predictability of the constraint could also play a role. One can assume that knowing in advance that a change is going to take place (predictable) allows one to prepare and anticipate it, contrary to the case where the change is sudden and unannounced (unpredictable). Therefore, we assume that adding a constraint will have an effect on the change of goal (H1). Furthermore, flexibility and metacognition are likely to be involved in goal choice. We tested the implication of individual flexibility and metacognition capacities in goal choice, especially when faced with an environmental constraint. Since flexibility allows one to adapt by considering changes in a situation when pursuing a goal, we hypothesized that the most flexible children would better adapt to changes in the environment by keeping their initial goal. As proposed by Sternberg (1990) and Sternberg and Powell (1983, p. 405) “flexibility in strategy or information utilization means that an individual knows when to change strategy or transfer information and when not do so” we distinguish two components of categorical flexibility: maintenance and switching. These two components have already been studied and highlighted (Maintenant et al., 2011, 2013).

The less flexible children would be more distracted by environmental changes that disrupt the task, and consequently would change their goal to a larger extent. Indeed, this fundamental EF contributes to the adaptation and control of one’s behavior by analyzing the situation from different angles. It provides an appropriate response to the situation: changing one’s goal when we are faced with an impasse or continuing with our initial goal if the change that occurred in the environment has no impact on the achievement of the task. In other words, cognitive flexibility allows us not to be disturbed by new constraints that do not prevent the initial goal, i.e., making use of proactive control. Moreover, the presumed superiority of the most flexible children should be observed more in the unpredictable change condition which involves the reactive control mode to a greater extent (H2).

Since metacognition allows for guiding, coordinating, controlling and/or modifying subordinate cognitive processes, it may also be involved in goal change. Based on the same reasoning as for flexibility, we hypothesized that the most metacognitive children should better adapt to constraints, by keeping their initial goals, especially in the face of an unpredictable constraint (H3).

Finally, we expected performance to change over time, due to the rapid development of self-regulation processes in young children. Hence, the different performances (goal change, flexibility, and metacognition) should increase at the second measurement point, 6 months after the first one (H4).

## 2. Materials and methods

### 2.1. Participants

Initially, 106 children (*M* = 4 years 4 months, *SD* = 3.85, rank 3 years 8 months - 5 years, 57 girls) participated. Their socio-cultural background was varied, as were the socio-professional categories of their parents (mixture of disadvantaged, modest, and favored populations). All had French as a native language. These children were all enrolled in preschool classes located in northern France. Teachers indicated that all the children in the sample were typically developing with no specific cognitive or learning difficulties. In this study, children were interviewed twice: in the fall (T1) and spring (T2) of the school year - which corresponds to the beginning and the middle of the school year in France. Six children provided incomplete data (change of school or absence from at least one test) and were therefore not included in the final sample. The analyses finally included 100 children (M = 4 years 4 months, SD = 3, 84; rank 3 years 8 months - 5 years, 54 girls).

### 2.2. Procedure

The school district gave its authorization and written consent was given to the parents, and children gave oral consent to participate and signed a consent form by circling the corresponding smiley. The ethics committee in behavioral sciences of the University of [Lille] validated the protocol. This study includes five phases: three with the jigsaw puzzle, one of which (the first) is with the metacognitive interview (McKI adapted), and two flexibility phases. The summary of the sequence of these phases is shown schematically in Table 1 below.

**Table 1:** Typical timeframe of study.

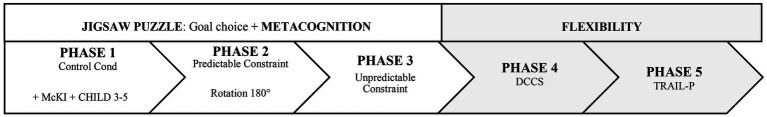					
	JIGSAW PUZZLE TASK			FLEXIBILITY TASK	
DAY	D1 Monday	D2 Tuesday	D3 Thursday	D4 Friday	D5 Monday
Phase	1 Control	2 Predictable constraint	3 Unpredictable constraint	4 DCCS	5 TRAIL-P
Material	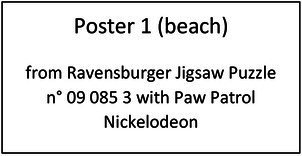 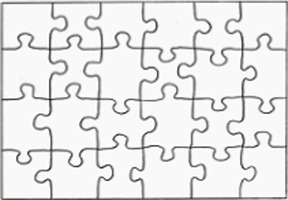 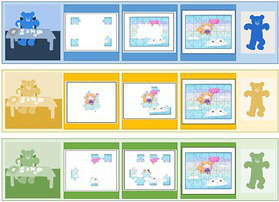 + METACOGNITION McKI adapted + CHILD 3–5	Help Support removal for half of the participants 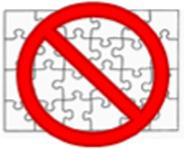 rotation of the puzzle for the other half 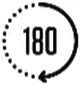 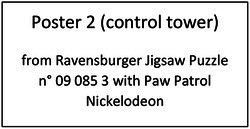 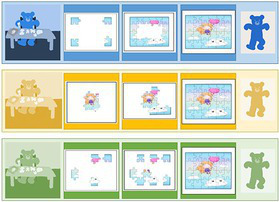	Rotation of the puzzle for half of the participants 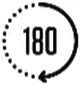 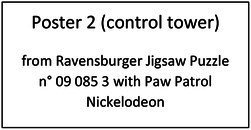 Help support removal for the other half 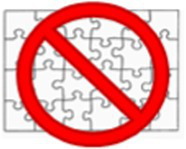 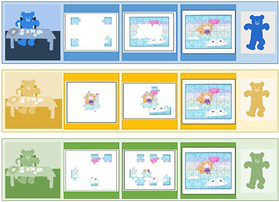	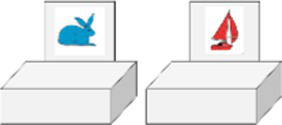 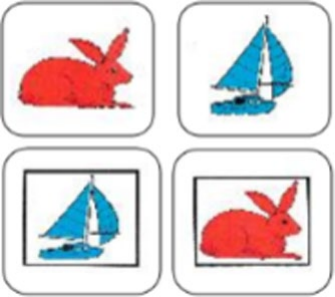	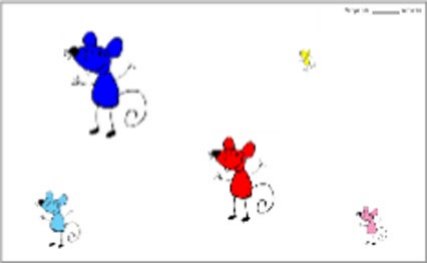 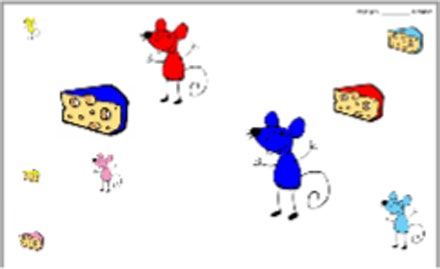
Scores taken	Goal chosen with GCTB: {1; 2; 3} before starting Score at McKI / 22 CHILD 3–5 score /66	Goal chosen with GCTB: {1; 2; 3}: before starting after the constraint (4 pieces placed)		Nb. of correct trials in phase 3 on board/12	Time (in sec) taken to make the board: - 1 (P1Control) - 2 (P2 Test 1)

Details of the five phases of the study. The original Ravensburger Jigsaw Puzzle images are available via the link: https://www.ravensburger.fr/produits/puzzle/puzzle-enfant/puzzles-2x24-p-des-museaux-efficaces-pat-patrouille-09085/index.html.

In order to follow the evolution over a school year, two measurement points were carried out: one in the fall at the beginning of the school year and the other in the spring, in the middle of the year, i.e., about 6 months later. For both measurement points, the five phases were conducted in their entirety each time and in the same order.

In the first three phases, the children were confronted with the jigsaw puzzle task but under three different conditions: {Controlled; Predictable; Unpredictable}. In the first phase (Day 1, control condition), children complete the jigsaw in the classical way within the 12 min allotted. No feedback is given, but the picture of the jigsaw and a help support representing the location of the pieces are available. In the other two conditions, a constraint is added: {Rotation at 180°; help support removal}. Thus, in the second phase (Day 2, Predictable Constraint condition), children are told that there will be a change. The jigsaw is presented to children again and it is explained to them that today they will do the jigsaw again but that this time, there will be a constraint (Rotate 180°). In the third phase (Day 3, Unpredictable Constraint condition) no guidance is given to children. They are simply told that they must show one last time how they do the jigsaw puzzle, but once they have put four pieces in place, the constraint is put in place (Remove help support for half of the participants). Since we have two change conditions (predictable/unpredictable), we made sure to also have two different types of constraints to be able to counteract a possible order effect. The allocation of participants was randomized so that half of the participants started with the rotation under predictable constraint condition and then the removal of the support under unpredictable constraint condition, and the reverse order was used for the other half of the participants.

#### 2.2.1. Jigsaw puzzle (phases 1 to 3)

All children passed through the three conditions in the same order (Control, Predictable, Unpredictable). The order of presentation of the three boards composing the *Goal Choice Teddy Boards* (GCTB) was counterbalanced randomly across children, but each child always had the same order. Similarly, the order of the type of constraint (180° rotation of the puzzle or removal of the help support) was randomly counterbalanced across children. The choice of the learning goal is recorded using the GCTB {Blue, contour = 1; Yellow, contiguity = 2; Green, random = 3} just before starting the jigsaw. To compare the impact of introducing constraints in the predictable and unpredictable conditions, in the last two phases, we surveyed this learning goal choice a second time just after the constraint in jigsaw puzzle completion (Rotation – Removal) was introduced. This allowed us to compare whether a change had occurred as a result of the change in the environment. In this way, we were able to determine whether or not children changed their learning goal after the change in the environment by comparing the chosen GCTB before and after the constraint. This gives us a binary categorical variable (1 = goal change; 0 = maintenance of the original goal) corresponding to what we will call the learning goal change scores.

#### 2.2.2. Flexibility (phases 4 to 5)

All children were given the two flexibility tests in the same order. The first flexibility test (DCCS) was offered to the children at phase 4 (Day 4). The score used was the score obtained in phase 3 with the border, i.e., the number of correct trials out of the 12 (Zelazo, 2006; Doebel and Zelazo, 2015). The order of the sorting criteria was counterbalanced. Half of the participants started with the color criterion and then the shape criterion and vice versa for the other half. The second flexibility test (TRAIL-P) was offered at phase 5 (Day 5), which closed the data collection at each measurement point. The score retained for this test corresponds to the time difference between boards 2 and 1, which is classically done with trail tests (Espy and Cwik, 2004).

#### 2.2.3. Metacognition (step 1)

The adaptation of the McKI is offered at the end of phase 1 (jigsaw puzzle done in control condition), once the child has finished his puzzle for the first time or at the end of the 12 min allotted. The CHILD 3–5 teacher questionnaire was distributed at the beginning of the study when the teachers were contacted to explain the study. The teachers were asked to complete the questionnaire within 2 weeks of the first stage at each of the two measurement points.

#### 2.2.4. Measurement point 2

For the second measurement point, 6 months after the first one, the procedure and the measures taken remain the same. The five phases are repeated: three with the jigsaw puzzle, one of which (the first) is with the metacognitive interview, and two of flexibility. All the children went through the three jigsaw puzzle conditions again, always in the same order (Control, Predictable, Unpredictable). However, in order to avoid a potential learning effect related to measurement repetition, two jigsaws were used, one for each measurement point. They had the same theme (Ravensburger® Paw Patrol theme) and were equivalent in terms of difficulty and number of pieces. The order of presentation of the jigsaws was randomly counterbalanced across measurement points. Thus, half of the participants started with jigsaw 1 and had jigsaw 2 for the second measurement point and vice versa for the other half of the participants. The order of presentation of the GCTBs remained the same as at the first measurement point for each child as well as the order of the type of constraint (rotate 180° or remove help support) in both conditions {Predictable; Unpredictable}. Thus, if children started with the removal in the predictable condition and then the rotation in the unpredictable condition at the first measurement point, they found exactly the same configuration at the second measurement point. The same scores as at the first measurement point were found for the three phases with the jigsaw, the metacognitive interview and the teacher questionnaire, as well as for the last two flexibility phases.

### 2.3. Materials and measures

#### 2.3.1. Choice of the learning goal

##### 2.3.1.1. Jigsaw puzzle task

The task used for this study involves a jigsaw puzzle suitable for 4-year-olds (Ravensburger® 6 × 4 collection; 26 cm × 18 cm) depicting familiar characters from a current popular cartoon (Paw Patrol®) that are staged as in Smiley and Dweck (1994). The jigsaw chosen for this study has 24 pieces (16 edges and 8 interiors) that are presented to children in bulk in the lid of the jigsaw box. The picture to be made is presented to children and they are questioned to check their understanding of the depicted scene.^1^ It is always left as a model for the child in a visible place on their workspace. A black and white help model showing only the location of the jigsaw pieces, but without the whole picture, is also left for children as a guide to complete the jigsaw (see Appendix A). The children are instructed to complete the jigsaw puzzle by assembling as many pieces as possible in the time allotted (12 min maximum). In order to evaluate the ability to complete the puzzle, the number of correctly placed pieces is noted (DV2) as well as the time it takes to complete the puzzle if the child stops before the 12 min are up (DV3 in seconds).

##### 2.3.1.2. The goal choice teddy boards

It should be remembered that since a jigsaw puzzle task has only one goal in itself, which is to put it back together correctly, we considered the specific procedure that the child intended to use to complete the jigsaw as constituting the learning goal. A review of the literature on the topic (see for example Smiley and Dweck, 1994; Sperling et al., 2000; Aral et al., 2012; Doherty et al., 2021) and a pretest with 14 kindergarten children (M = 4 years 9 months; SD = 2.95; rank 4 years 6 months - 5 years 3 months, 9 girls) with an observational grid replicating typical jigsaw puzzle completion behaviors, allowed us to distinguish three main procedures: the contour procedure, the adjacency procedure, and the random procedure.

The contour procedure, the most elaborate, consists of relying jointly on the spatial structuring of the scene and the shape of the pieces. This procedure involves looking at the shapes of the pieces to start with the corners and edges, and then completing the interior of the jigsaw. The adjacency procedure at the intermediate level consists of relying on figurative cues that identify the elements of the visual scene (shape and color of the characters and the setting). This procedure involves looking primarily at the model, beginning assembling a particular character, and then trying to complete it by looking for the pieces to put around it. The random procedure, the least elaborate, consists of completing the jigsaw at random. It involves taking the pieces without any predetermined plan and then trying to put them together.

**Figure 1:**
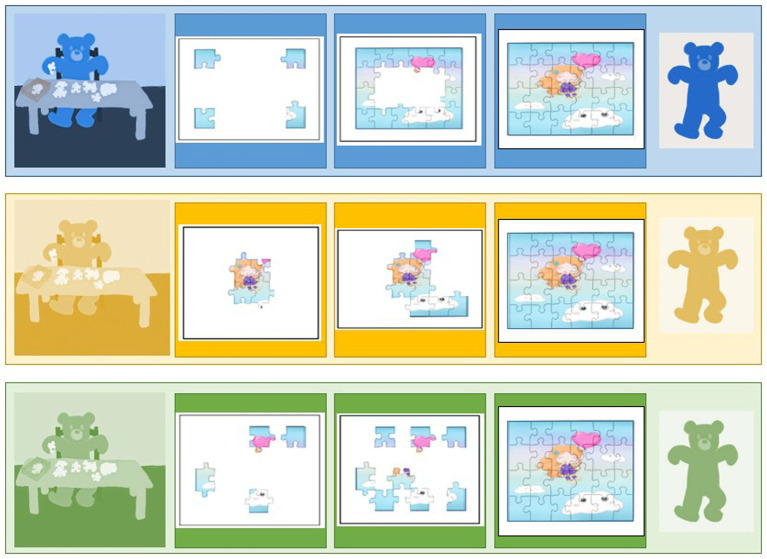
Representation of the Goal Choice Teddy Boards. The real size corresponds to 6 x 29,7 cm, for each one. The original version used in the study was made with pictures of the steps of making a jigsaw puzzle with the Ravensburger Puzzle 4+ N° 08 993 2 with the character Nihao Kai-Lan from Nickelodeon. For a question of authorization of the image rights, an adapted version was carried out for the present article with a free image carried out by Tarisha of Pixabay being close to the initial one.

To help the understanding of the instruction and to guide the choice of the goal explicitly and concretely, we built a specific tool: the Goal Choice Teddy Boards (“GCTB”; reproduced in Figure 1). The GCTB is composed of three boards illustrating the three procedures by a three-step schematization, repeated in colored vignettes. Indeed, it seems that the use of illustrated information helps in the realization of jigsaw puzzles and that this skill particularly develops during the preschool period (Doherty et al., 2021). Each of the procedures corresponds to a specific color teddy bear to which we refer to simplify the process. The contour procedure is shown with the Blue Teddy Board, the adjacency procedure with the Yellow Teddy Board and the random procedure with the Green one.

For data coding, we adopted a basic numerical code to match a number to each board, i.e., to each procedure: Blue Teddy Board, contour procedure = 1; Yellow Teddy Board, adjacency procedure = 2; Green Teddy Board, random procedure = 3. Just before the child begins, the experimenter explains that there are three procedures for making a jigsaw.

The GCTB is used as a medium to explain these procedures to the children. The children are given enough time to observe the boards and are then asked how they plan to complete the jigsaw puzzle. The procedure chosen by children is the dependent variable “goal choice” (DV1). The boards are then hidden and the timer is started. The children have 12 min maximum to assemble as many pieces as possible.

#### 2.3.2. Cognitive flexibility

We used two flexibility tests in order to access the respective roles of conceptual flexibility (DCCS) and attentional switching (TRAIL-P). Although these two types of processes both refer to the EF of cognitive flexibility, the underlying processes appear to be different and may play distinct roles in SRL, particularly in adapting to changes in the environment during goal choosing. The use of these two tests should provide information on the specificities of conceptual flexibility and attentional switching concerning goal choice but also on the links between them. Furthermore, the degree of agreement between the two flexibility tests used here has never been tested to our knowledge. It is important to determine the extent to which performance on the two tests correlates with each other, which will allow us to estimate the degree of overlap between the processes involved in these tests.

##### 2.3.2.1. The dimensional change card sort

**Figure 2:**
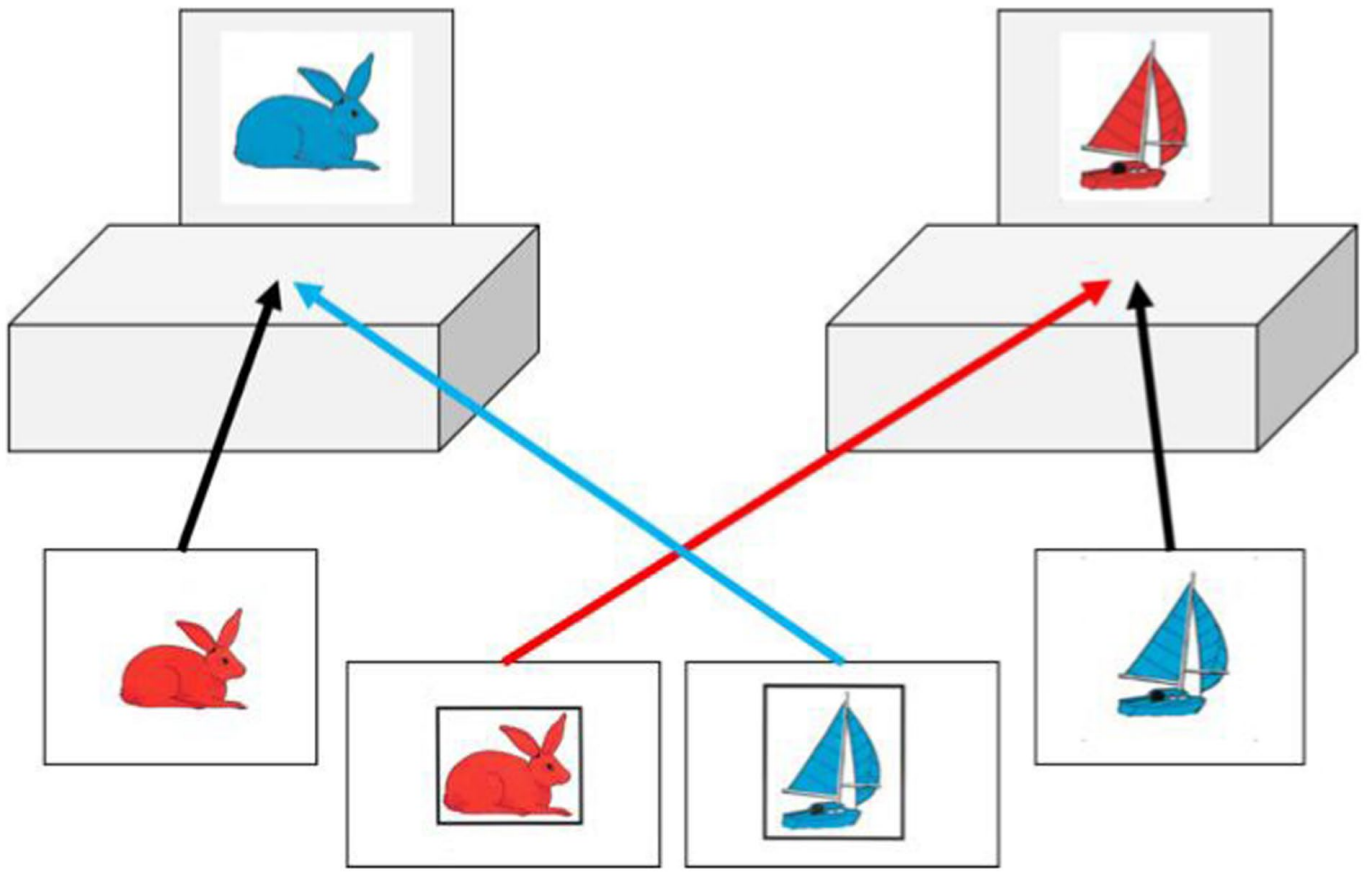
Schematic illustration of the DCCS Phase 3 of border version inspired by Chevalier and Blaye (2006) and Blaye (2021). Notes: Figure Black arrows correspond to a correct sort for the “shape set” while colored arrows correspond to a correct sort for the “color set” (cards with a black border).

We began by using the most widely used test for measuring flexibility in preschoolers: the Dimensional Change Card Sort (“DCCS”, Frye et al., 1995). This test is presented as a card sorting game with alternating sorting rules. Test cards representing an object (e.g., a red rabbit or a blue boat) must be sorted into two boxes on which a target card is fixed (a blue rabbit or a red boat) and alternated according to a criterion, shape or color, depending on the sorting rule given (Doebel and Zelazo, 2015). We chose a slightly more difficult version: the DCCS border version (Zelazo, 2006), reproduced schematically in Figure 2 below. It includes a third and final phase, where new cards with a black border surrounding the drawn item (rabbit or boat) are introduced. The border symbolizes a specific game and the rules that go with it: the color game.

Thus, at each trial, children must choose to apply the correct sorting rule according to color or shape. As in the standard version, the rules and the change of rules are explicitly announced and systematically repeated before each trial to limit the burden on working memory. No feedback is given to children. This third phase includes 12 trials and the score is calculated only on these 12 trials (DV2). This border version is only offered if children have obtained a total score in Pre and Post Switch ≥10, which is equivalent to obtaining at least 5/6 in Pre Switch and 5/6 in Post Switch (two errors maximum). Otherwise, they get a score of 0/12. Insofar as a ceiling effect is often found with the classic version of the DCCS from age 4, this more complex framework version seemed more appropriate here. In addition, to pass the test, children must be able to change the way they sort the test cards in response to the change in instruction. This test refers more to the conceptual aspect associated with flexibility, reporting on the increase in flexibility through the development of comprehension and causal reasoning skills from conditional if-then rules (Chevalier and Blaye, 2006; Doebel and Zelazo, 2015).

##### 2.3.2.2. The preschool tracking test

In order to apprehend the perceptual aspect of flexibility, it is preferable to turn to trail tests: this is why the second test used in this study is the TRAIL-P © Test taken from the BEFEX-P 4.0 battery by Monette and Bigras (2015). This is a paper-and-pencil format test with mice of different sizes and colors that the child must connect as quickly as possible. Each one has its own color and size and each one has a cheese of the same color and size as it. TRAIL-P consists of three test boards and three practice boards. In the first condition (control), only the mice are present and the child must connect them in ascending order, from the smallest mouse to the largest. In the second condition (test board 1), children must again link the mice in ascending order, but this time, there are also pieces of cheese. They have to alternate with the corresponding cheese of the same size and color before moving on to the next mouse. In the third condition (test board 2), children must perform the same task as in the second condition, but distracting stimuli such as a rabbit, carrot, monkey and banana are also present. Before each test run and for each condition, a demonstration and a practice trial take place. Regardless of the condition, errors are immediately reported and corrected by having children start over from the last correct location. The scores reported are, for each condition, the time taken to complete the task and the number of errors made. The degree of perceptual flexibility component is estimated by the difference (in seconds) between the time taken to execute the test board 1 and the time taken to execute the control board (DV3).

These two preschool cognitive flexibility tests, which are among the most widely used, are expected to predict children’s goal choice. Using both tests may provide details on both the specifics of each process, conceptual flexibility and attentional switching, in goal choice in the face of an unstable environment with constraints. In addition, the TRAIL-P, which is an attentional switching task, reflects a more perceptual dimension of flexibility that should be more closely associated with the change of goal and the influence of the conditions of realization of the jigsaw puzzle. For this reason, this attentional switching task might be a better predictor of goal change than the DCCS task, which requires mastery of a more complex rule system reflecting a more conceptual dimension of flexibility. Moreover, beyond the specificities of each process, using two measures should also provide evidence of how these different facets of cognitive flexibility might be related.

#### 2.3.3. Metacognition

Following the same reasoning as for measuring flexibility, we used two metacognition tests in order to account for the two components of this cognitive function and to clarify its contours. To access broad metacognitive skills, we chose the Checklist of Independent Learning Development for children ages 3 to 5 (CHILD 3–5; Whitebread et al., 2009), a teacher questionnaire. To access metacognitive knowledge, we chose the Metacognitive Knowledge Interview (McKI; Marulis et al., 2016). These different components of metacognition could play distinct roles in self-regulation, particularly in adapting to changes in the environment during goal setting. Using both should provide information about their specificities concerning goal choice as much as on how they are related. Furthermore, the degree of agreement between these two metacognition tests has never been tested to our knowledge. However, it is important to determine the extent to which performance on the two tests is correlated with each other, which will allow us to estimate the intensity of the overlap of the processes involved in these tests.

##### 2.3.3.1. The checklist of independent learning development

First, we used the CHILD 3–5 by Whitebread et al. (2009), which we translated. It is presented as a questionnaire for teachers, composed of 22 items allowing assessment of the child’s behavior through a 4-point Likert scale (Never = 0; Sometimes = 1; Regularly = 2; Always = 3 points). The maximum score is 66 (DV4). The CHILD 3–5 is an indirect measure of metacognition that provides an account of overall and broad metacognition skills through the teacher’s judgment.

##### 2.3.3.2. The metacognitive knowledge interview (McKI)

The second tool we used was inspired by the work of Marulis et al. (2016) as we adapted their McKI to our jigsaw task. The McKI is a post-task interview that involves making three (or four for the best) constructions of increasing difficulty by assembling interlocking plastic elements. The interview focuses on the activity performed through a series of 11 questions that concern both the children’s knowledge specific to the task and the children’s knowledge about themselves and this task (metacognitive knowledge). For each of the 11 questions, the score can vary on a scale from 0 to 2 points, where a score of 0 = not at all metacognitive response, 1 = partially metacognitive response, 2 = appropriate metacognitive response. The authors also provide a McKI scoring guide including sample scores to assist in accurate scoring. In the original English version from Marulis et al. (2016), the maximum score is 22, which makes it possible to refine the elements concerning the specific metacognitive knowledge of children when faced with a precise task (“Wedgit”: plastic construction elements). For our study, we used the same framework, which we specifically adapted to the task of completing a jigsaw. This interview is presented to the children as a set of questions to help the experimenter better understand how the child completed the jigsaw, these questions do not include wrong answers. The maximum score is the same as in the original version: 22 (DV5). The McKI involves the child’s metacognitive knowledge about completing a jigsaw, thus reflecting a more conceptual dimension of metacognition.

With the same reasoning as above, these two preschool metacognitive measures are expected to predict children’s goal choice. Using both tests may provide details on both the specifics of each dimension, the broad metacognitive skills and the metacognitive knowledge, in goal choice in the face of an unstable environment with constraints. In addition, it can be assumed that the CHILD 3–5 which gives a better account of the child’s ability to mobilize coping strategies, and thus reflects a more procedural dimension of metacognition should be more closely associated with the change of goal and the influence of the conditions of the jigsaw completion. For this reason, the CHILD 3–5 may be a better predictor of goal choice than the McKI, reflecting a more conceptual dimension of metacognition Moreover, beyond the specificities of each process, using two measures should also provide evidence of how these different facets of metacognition might be related in early childhood.

## 3. Results

### 3.1. Experimental design and preliminary analyses

The study is broken down into five phases (Table 1): three with the jigsaw puzzle including 1 with metacognition, and two flexibility phases. An experimental design 2 (Measurement Point: 1 or 2) * 3 (Condition of change: Predictable, Unpredictable, Control) was used. Each child provided 9 different scores (Goal choice: one measure in control condition, two measures in the two conditions with constraints: one before change and one after change; Flexibility 2 measures; Metacognition 2 measures) at each measurement point, for a total of 1,800 exploitable individual scores that were used for the statistical analyses. These statistical analyses were performed with Statistica 13.3 ©.

A Preliminary data analysis allowed us to test for possible effects of order of presentation, constraints, order of jigsaws, and gender, on jigsaw puzzle completion performance. No effect was significant. Thus these factors were not included in the following analyses.

### 3.2. Influence of conditions on the realization of the jigsaw puzzle on the goal choices

#### 3.2.1. Constraints of realization of the jigsaw and goal

**Figure 3:**
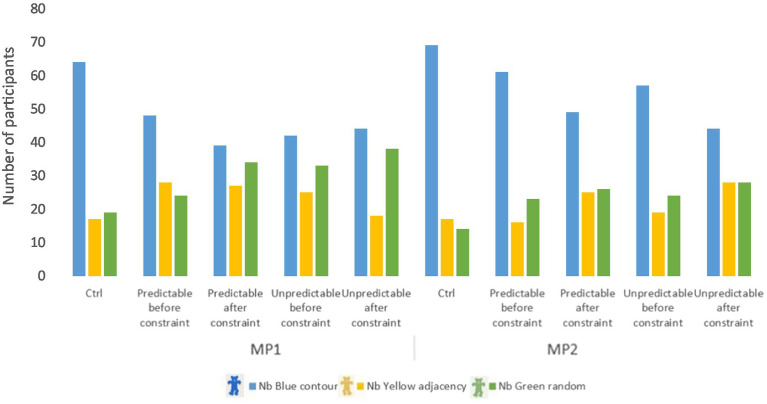
Evolution of goal selection collected with GCTBs by measurement point and conditions, before and after constraint.

We tested the possible influence of the jigsaw puzzle realization condition (Control, Predictable, Unpredictable constraint) on goal change. Figure 3 below represents the evolution of participants’ goal choices as a function of the jigsaw completion constraints and the measurement point. We first considered the goal chosen before the constraint for the Predictable and Unpredictable conditions.

We can see that the contour strategy (Blue Teddy) is always the most chosen one, regardless of the condition or the measurement point. We wanted to check if this choice was voluntary and we performed Pearson’s Chi 2 tests according to the three conditions of the jigsaw puzzle, before and after the constraint, and for the two measurement points. The results show that the distribution of choices of Teddy procedures is not random at the first measurement point: pre-constraint *χ^2^*(dl = 4) = 11.78, *p* < 0.05 and post-constraint *χ^2^*(dl = 4) = 16.69, *p* < 0.01. The results for the second measurement point are not significant pre-constraint, *χ^2^*(dl = 4) = 14.31, *p* =. 35, *ns*, but reveal a non-random distribution of choices after constraints, *χ^2^*(dl = 4) = 14.31, *p* < 0.01. Thus, the distribution of choices for the Teddy procedure (learning goal choice) differs under the conditions of jigsaw completion.

#### 3.2.2. Predictability of constraint and goal choice

**Figure 4:**
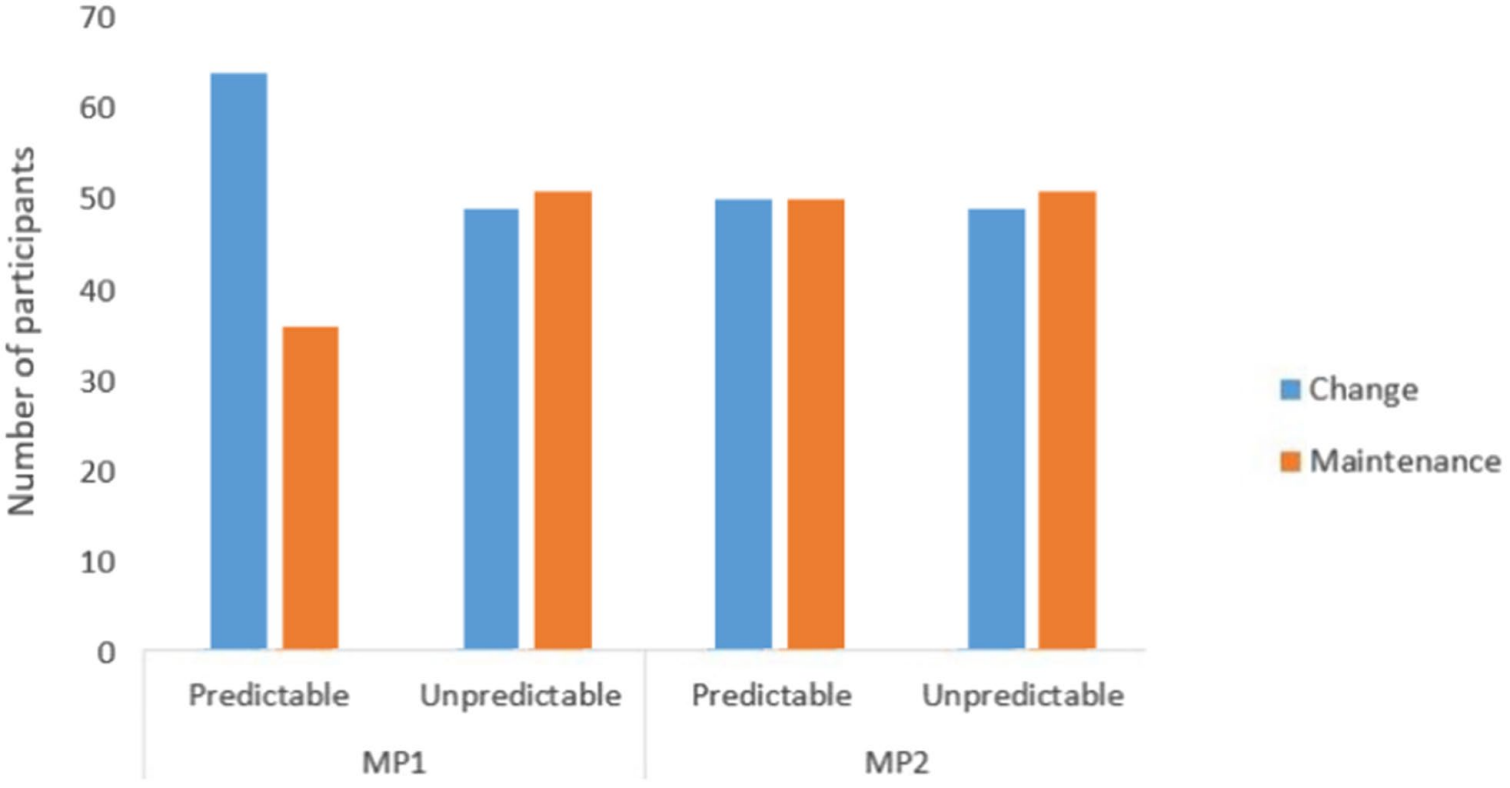
Evolution of goal change after introduction of an environmental constraint, depending on the measurement point and the predictability of the constraint.

In order to test whether a change in the task conditions can cause children to change their goal, we compared the choice of learning goal (contour, adjacency, or random procedure), before and after the introduction of the constraint, for the two constrained conditions {predictable; unpredictable} and for the two measurement points. We thus obtained a binary categorical variable {0 = Maintain Goal; 1 = Change Goal}. We calculated the number of participants who maintained their initial learning goal after the introduction of the constraint, and the number of participants who changed their learning goal after the introduction of a constraint, for both types of constraint and both measurement points. The evolution of goal changes as a function of measurement point and constraint predictability is shown in Figure 4.

At MP1, under the Predictable constraint condition, children who changed their goal after constraint are almost twice as many as those who maintained their goal, *n* = 64 and *n* = 36, respectively. In the Unpredictable constraint condition, the gap narrows considerably to almost equal numbers (*n* = 49 change vs. *n* = 51 retention). At MP2, the numbers are identical under the Predictable constraint condition (*n* = 50), and nearly identical under the Unpredictable constraint condition, *n* = 49 and *n* = 51, respectively. Over the course of 6 months, children were more able to maintain their initial learning goal rather than change it, as they tended to do at the first measurement point in the predictable condition.

Cochran tests were used on the goal change score (0 = Goal maintenance; 1 = Goal change) in the predictable vs. unpredictable constraint condition, for both measurement points. A significant difference appeared at MP1 [*Q* (dl =1) = 6.43, *p* < 0.05] but not at MP2 anymore [*Q* (dl =1) = 0.04, *p* = 0.85, *ns*]. Thus, at MP1, announcing an environmental change in jigsaw completion (predictable constraint) resulted in more children changing their learning goal in the Teddy procedure than children retaining their initial goal, this difference not being found after an unannounced environmental change (unpredictable constraint), contrary to our expectations. In MP2, this effect was not found in either change condition. These results highlight the sensitivity of children to change when they were encountered at the beginning of the school year, and an attenuation of this sensitivity a few months later.

### 3.3. Influence of flexibility on the goal choice

Descriptive statistics of the different dependent variables related to flexibility and metacognition are summarized in Table 2.

**Table 2:** Descriptive statistics of the dependent variables of study.

Variables	MEASURING POINT 1				MEASURING POINT 2			
	*M*	Minimum	Maximum	*SD*	*M*	Minimum	Maximum	*SD*
Flexibility								
DCCS (/12)	4.03	0	10	3.41	6.09	0	12	2.56
TRAIL-P (sec)	62.92	– 174	360	82.38	37.13	– 33	297	49.72
Metacognition								
McKI (/22)	8.88	0	16	3.39	11.36	3	18.50	3.42
CHILD 3–5 (/66)	36.46	6	59	10.45	44.55	14	66.00	12.70

Logistic regression (Logit) analyses allowed us to test the relationship between flexibility and goal change as a function of constraint predictability (Table 3).

**Table 3:** Effect of flexibility scores on goal change scores (logistic regression).

Predictor	MP1				MP2			
	Predictable condition		Unpredictable condition		Predictable condition		Unpredictable condition	
	Wald Stat	*p*	Wald Stat	*p*	Wald Stat	*p*	Wald Stat	*p*
DCCS	0.01	0.91	0.00	0.98	0.95	0.33	5.79	0.02*
TRAIL-P	0.00	0.96	3.64	0.057♦	1.07	0.30	0.01	0.92

Significance levels: *p < 0.05; ♦ < 0.10.

At the first measurement point (MP1), only TRAIL-P performance showed a significant trend in predicting goal change in the unpredictable condition (*W* = 3.64, *p* = 0.057) but no longer thereafter, nor in the predictable condition. At the second measurement point (MP2), only DCCS performance was found to be significantly predictive of goal change in the unpredictable condition (*W* = 5.79, *p* < 0.05). Thus, individual flexibility abilities were involved in coping with change, but only when the latter was unpredictable. Moreover, perceptual flexibility measured with TRAIL-P was significant only at MP1, whereas conceptual flexibility measured through DCCS was significant only at MP2, speaking to a difference in the developmental agendas of both kinds of flexibility.

### 3.4. Influence of metacognition on the goal choice

As previously with flexibility, we also tested the relationship between metacognition and goal change (Table 4). Logistic regression analyses on CHILD 3–5 scores revealed a single significant effect at MP1 in the unpredictable condition (*W* = 4.03, *p* < 0.05) but not in the predictable condition (*W* = 0.40, *ns*). At MP2, no effect was significant (in the predictable condition: *W* = 0.005, *ns*; in the unpredictable condition: *W* = 0.08, *ns*). Thus, at MP1, children with the highest CHILD 3–5 scores maintained their goal the most in the unpredictable constraint condition. Similar analyses with adapted McKI scores as predictors revealed nothing at MP1 (in predictable condition: *W* = 1.63, *ns*; in unpredictable condition: *W* = 0.11, *ns*). At MP2, a significant effect appeared in the unpredictable condition (*W* = 5.56, *p* < 0.05) but not in the predictable condition (*W* = 0.99, *ns*). Thus, at MP2, the most metacognitive children according to the McKI were also those who maintained the most of their goal in the unpredictable constraint condition.

**Table 4:** Effect of metacognition scores on goal change scores (logistic regression).

Predictor	MP1				MP2			
	Predictable condition		Unpredictable condition		Predictable condition		Unpredictable condition	
	Wald Stat	*p*	Wald Stat	*p*	Wald Stat	*p*	Wald Stat	*p*
CHILD 3–5	0.40	0.53	4.01	0.045*	0.01	0.94	0.08	0.78
McKI	1.63	0.20	0.11	0.74	0.99	0.32	5.56	0.02*

Significance levels: *p < 0.05.

## 4. Discussion

The main objective of this study was to determine factors that influence goal choice in preschoolers. Specifically, we tested whether the addition of a constraint to the completion of the jigsaw puzzle impacted learning goal choice (H1). Further to this, we investigated the influence of cognitive flexibility (H2) and metacognition (H3) on the ability of 4-year-old children to choose a learning goal. We also tested the influence of change over time, by comparing performance at a 6-month interval (H4). Our hypotheses have been validated and we will now return in detail to each of them.

### 4.1. Determinants of learning goal choice

#### 4.1.1. Constraints

In our study, children were confronted three times with a jigsaw puzzle, two of which were with constraints that involved adaptation. First, we assumed that the conditions under which the jigsaw puzzle was completed may impact children’s goal choice (H1). In particular, we compared two conditions: a predictable change, where the constraint to complete the jigsaw is announced beforehand; and an unpredictable change, where the constraint occurs without being announced beforehand. Our results allow us to validate this first hypothesis. Indeed, children did not choose the jigsaw completion procedure at random, this choice varying according to the conditions of the jigsaw completion and the measurement point. The creation of a specific and explicit tool for identifying solving procedures, such as the one we have created (GCTB), appears to be an interesting and relevant way to study complex processes like choosing a learning goal in young children.

However, and contrary to our expectations, it seems that it is the addition of a predictable constraint that disturbs children more and pushes them to change their learning goal. At age 4, regarding the impact of conditions on goal choice, the results showed a significant effect of introducing a constraint to complete the jigsaw puzzle, compared to the control condition: children tended to change their learning goal more in the predictable change condition and seemed to be more able to maintain their initial learning goal in the unpredictable condition. The difference was no longer significant at age 4.5, where children who changed their learning goal were no more numerous than those who maintained it, whether in the predictable or unpredictable condition. We expected this to be more the case in the unpredictable condition but in this condition, there was no longer significant difference between children who maintained and those who changed their learning goals.

This can be explained in relation to the Forethought Phase of SRL (Zimmerman, 2013). Indeed, in this preparation phase, learners analyze the task and build a strategic plan to achieve the fixed goal. Choosing a goal implies a precise analysis of the task to be accomplished, as well as taking into account the information to be gathered and the plans to be developed to reach it (Galotti, 2005). In this study the children were faced with a new learning task since they did not yet master the procedures for completing a jigsaw. Thus, the procedure was in itself a learning goal - a specific skill that the child does not yet have, and which he/she aims to acquire through the task. Also, the addition of an explicit constraint before embarking in the activity, may have pushed the children to anticipate a change in their planning as a kind of alert (*‘be careful*, *there will be a change’*), effectively pushing them to change their goal by taking into account this change in the environment.

This result can also be interpreted according to the developmental model of cognitive control proposed by Braver (2012). Indeed, young children tend to adopt a reactive mode of control that is based on the cues available in the environment (Chevalier et al., 2017). Thus, announcing a change can be thought of as a new instruction about the task, which prompts children to change their goal. Presumably, announcing that there is going to be a change disrupts information processing and interferes with task performance. The child may think “*Oh look out! Soon I will have to do it backwards; how am I going to do it?*” and thus be more focused on his thoughts rather than on the jigsaw itself. This is consistent with the findings of Blakey et al. (2016) who showed that preschoolers can be distracted by task-irrelevant information during a rule change, even when a response conflict is completely absent.

Moreover, this developmental difference in children’s responses between the first measurement point (when children are 4 years old) and the second measurement point (when children are 4.5 years old) could be explained by individual capacities for flexibility and metacognition. Indeed, our results show that these two cognitive functions predict learning goal change at both time points.

#### 4.1.2. Flexibility

Concerning our second hypothesis, we assumed that flexibility would predict goal choice (H2). We expected that the most flexible children would better adapt to changes in the environment by not changing their learning goal, especially in the face of an unpredictable constraint (H2). This second hypothesis is validated. The predictability of a change thus seems to be a determining factor which is linked to flexibility capacities in young children. The results show an influence of flexibility on the choice of a learning goal, and only in unpredictable conditions as we assumed. Since the predictable condition did not give any significant result, all the results discussed below are therefore only for this unpredictable condition. Moreover, the results are complementary depending on the flexibility measurement tools used. Thus, at age 4 only the results obtained with the TRAIL-P were predictive of the choice of the learning goal. The TRAIL-P refers to perceptual processes, with alternation from conditions to link the different elements (condition 1 a single category: mice; condition 2 alternating two categories: mice and cheeses). This test calls on the children’s ability to switch their attention between mice and pieces of cheese, and to avoid being distracted and attracted by intruders. It also refers to reactive flexibility, which relies on environmental cues to redirect attention to previously ignored properties.

At age 4.5, it is rather the conceptual flexibility measured through DCCS that significantly predicted the choice of the learning goal. The DCCS involves thinking about a complex system of hierarchically integrated “if-then” rules and thus refers to a high level of cognitive complexity (Zelazo et al., 2003). This conceptual understanding of the task implies anticipating several points of view on the task in order to choose the optimal strategy, independently of the signals from the environment. It thus implies more spontaneous flexibility which manifests itself by a disengagement from the action, this disengagement representing a change which is not constrained by the situation, but on the contrary, anticipated by the individual (Clément, 2006, 2021).

The results suggest that the efficiency of this conceptual component of flexibility needs more maturation since it takes until the age 4.5 to discriminate between children. In the unpredictable condition, the change is announced just before implementing the constraint. Thus, it requires the child to adapt quickly and it mainly involves the reactive control mode depending on environmental signals. Indeed, the constraint that occurs suddenly in an unpredictable way can be interpreted as a new signal that guides the task. It can be assumed that the cost generated by this unexpected constraint disturbs information processing and leads the less flexible children to be more influenced by this signal, and in turn, to change their learning goal. In their meta-analysis of the DCCS, Doebel and Zelazo (2015) have indeed shown that verbal labeling of a single dimension or of multiple dimensions has an overall facilitative effect, but it can sometimes have a disruptive effect. This would occur because it reinforces the active representation of rules in pre-switching, which in turn reinforces the latent representations of these rules (Yerys and Munakata, 2006). Similarly, emphasizing the conflict between rules by focusing on the end of the first game and on the fact that a new and different game must be played, tends to increase flexibility. Indeed, this focus would increase the child’s reflection on the hierarchical structure of the task and the incompatibility of the rules, thereby eliciting the activation of top-down control *via* the perception of conflict. Beyond that, the different existing variations of this test have also shown the importance of the salience of the dimension after the change of the sorting rule on switching performance, and of the degree of spatial separation of dimensional values. Children benefit the most when post-switch salience is increased and the dimensional values are completely spatially separated. In contrast, feedback, although increasing explicit awareness of the pre-switch rules, has no effect. This is again indicative of a tendency to adopt a reactive control mode influenced by environmental cues, a control mode characteristic of young children.

#### 4.1.3. Metacognition

Our third hypothesis concerned the possible influence of metacognition on learning goal choice (H3). This hypothesis is validated since our results show that metacognition significantly predicted the scores on goal choice. Again, it was only the case under unpredictable constraints though, and this is why we will discuss the results below only for this condition. Differences appear according to the dimensions of metacognition considered. Indeed, at age 4, the influence of metacognition on the choice of goal emerges significantly with the CHILD 3–5, which measures broad metacognitive skills. At age 4.5, it is the specific metacognitive knowledge measured by the McKI that gives a significant effect. These results show that both sides of metacognition are involved in choosing a learning goal in the face of an unpredictable change in the environment, in order not to be diverted from the intended goal.

Moreover, these results seem to be complementary to those revealed by the flexibility tools, suggesting the existence of links between flexibility and metacognition as currently envisioned theoretically. Thus we provide new evidence for the existence of correlations between these two critical cognitive functions, as has been shown in the past (Roebers, 2017; Marulis et al., 2020). Our results support this, by showing links between flexibility, metacognition, and environmental changes. These links exist whether the change is predictable or not, and at an age that is crucial for the development of these cognitive functions.

### 4.2. Developmental evolution

Finally, in order to account for the development of goal choosing capacities, our last hypothesis involved showing changes in children’s performance between the two measurement points (H4). Overall, we have shown that the different performances evolved between the two measurement points. Children tended to change their learning goal less under predictable constraints at age 4.5, thus becoming less perturbed by changing environmental cues. To highlight the progression and developmental aspect of our results, we can compare the performance at both measurement points. If we look at the evolution between the two measurement points, we can see that the ability to choose a goal and maintain it in young children changes slightly. Children are less likely to change their goal in the predictable condition as they were likely to do so at age 4. Thus, at age 4.5, as many of them change their goal as maintain their initial goal. The results suggest that when the task is first presented at age 4, children process the information from the environment at a perceptual level (reactive mode of control), and then at age 4.5 they process it at a more conceptual level (proactive mode of control). This interpretation is in line with representational re-description (Karmiloff-Smith, 1994) which implies the passage from an implicit level based on external information, encapsulated in the procedure (behavioral mastery), to a more explicit and more flexible level. Indeed, as children grow older, they tend to better regulate their behavior in complex situations (Diamond, 2013; Thaqi and Roebers, 2020), adopting a proactive mode of control in which they can better anticipate the effects of their actions (Chevalier et al., 2017). This confirms and extends results reported by Bordes et al. (2007) in one study that examined the development of 6-, 8-, and 10-year-olds’ ability to adapt to an unexpected goal change in a drawing memory task. In that study, children were told that they had to memorize the drawing to be able to redraw it later. In one condition (congruent goal) this was indeed the case but in another condition it was not, since they had to find the correct drawing among others (incongruent goal). Bordes et al. (2007) have shown that adaptation to an unexpected goal change increases between 6 and 8 years of age but is not really operative until around 10 years of age. A slow developmental increase in the capacity to adapt to an unexpected goal may explain the lack of effect in the unpredictable condition in our study.

Participants in our study also improved their performance on flexibility and metacognition tests between both measurement points. Our results also provide a better understanding of the developmental trajectory of flexibility: under unpredictable constraints, perceptual flexibility seems to be more mobilized, and it seems to appear earlier since we observe a significant effect only at 4 years old. The conceptual component of flexibility seems slower to develop, since a significant effect is observed only at age 4.5.

These results also demonstrate the specificity of flexibility assessment tools, which allow distinguishing the processes underlying flexibility as suggested by previous studies (Doebel and Zelazo, 2015; Clerc et al., 2021): the DCCS allows considering conceptual components of flexibility while the TRAIL-P would account for more perceptual aspects. Thus, it seems that flexibility abilities evolve in a few months, and that their implication changes according to the type of tool used to measure these abilities. This is consistent with the results of Ebersbach and Hagedorn (2011) who were able to show that flexibility is invested differently depending on the type of task chosen, in a spatial drawing study with 7-and 11-year-olds.

Finally, in front of an unpredictable constraint, we were able to show that metacognitive skills are more involved in the choice of a learning goal at age 4. At age 4.5, it is the metacognitive knowledge that takes over and significantly predicts the choice of goal. This is in line with the results observed with the flexibility measures, confirming that initially, it is more the perceptual and procedural aspects that influence the choice of a learning goal in the face of an unexpected change, whereas 6 months later, it is the conceptual aspects that make the difference in explaining goal choice. These results re-emphasize the importance of executive development in supporting metacognitive development and promoting activity control. Flexibility and metacognition can then be seen as precursors of self-regulation of learning.

### 4.3. Limitations and perspectives of the study

For this study, we chose to use the completion of a jigsaw puzzle. We were able to show that with an adapted support (the GCTB), a child can choose a learning goal explicitly. This allows us to respond to the lack of specific tools in the research on SRL (Winne, 2005; Azevedo, 2009). Moreover, the GCTB allowed us to follow the child’s progress as closely as possible, since we suggested that they use them at different moments of the SRL process during the completion of the task, and not only at the beginning. Here we were especially interested in the first stage of SRL, the Forethought Phase, which includes choosing a learning goal. SRL is a dynamic process though, which comprises several steps. Thus, even if a child chooses a particular learning goal, there is no guarantee that he/she will actually pursue it. In this study, we did not verify that the child completed the puzzle with the procedure that he/she had chosen to learn at the beginning of the task. It would be necessary in future studies to verify whether children actually carry out the chosen procedure to complete the jigsaw. The GCTB could even be used as an aid to help the child remember its intended procedure, since the different procedures are visible on the board. Such help would be valuable given that the procedure for completing the jigsaw is in itself a learning goal in such young children. In addition, an adult may be present to further accompany children in their procedure and guide them (Robson, 2016). This offers interesting leads towards the world of education and the role that the teacher could play.

Another limitation is about longitudinal follow-up. We followed the evolution of the children over a school year by taking two measurement points separated by 6 months: the first at the beginning of the school year, the second in the middle/end of the school year. It would be interesting to take more measurements at more regular intervals, and to broaden the age span of the participants. Indeed, longitudinal studies are rare, and even more so in young children, although it is recognized that the period between 2 and 6 years of age is decisive for the development of SRL, EF and metacognition (Roebers, 2017; Dörr and Perels, 2020; Marulis et al., 2020).

One step forward would be to look more deeply into the links and overlaps between developing EF and metacognition in order to determine more precisely their respective roles and shares in the evolution of children’s skills. Recent work suggests that EF and metacognition share parallel developmental trajectories and show a dynamic relation across development (Bryce et al., 2015; Roebers, 2017; Marulis et al., 2020; Pennequin, 2021). EF maturation is thought to be critical to the development of metacognitive abilities (Bryce et al., 2015; Roebers, 2017). However, studies are still rare and evidence to support this hypothesis is lacking.

## 5. Conclusion

This study aimed to better understand the links between SRL, flexibility and metacognition in children aged 4 to 5 years. We were able to show that the conditions under which a jigsaw is completed can influence the choice of a learning goal in young children. It appears that the addition of a predictable constraint can push young children to change their learning goal during the task. Before age 4.5, children tend to be disturbed by changes in the environment. This result has been shown thanks to the creation of an original tool, the GCTB. This reinforces the reflection launched by many authors (notably under the impulse of the work of Whitebread et al., 2009, or more recently of Marulis et al., 2016) regarding the tools and protocols used with a young population.

On the other hand, the influence of a constraint in the choice of the learning goal was not found in the unpredictable condition, nor in the second measurement point. In these other conditions, we were able to show that goal choice was influenced by flexibility and metacognition, which can be viewed as precursors of SRL (Follmer and Sperling, 2016). Our results also suggest that there are links between these two cognitive functions that it would be relevant to study and take further.

Finally, this study also allowed us to emphasize the specificities of two tools measuring flexibility and two others measuring metacognition, offering the possibility of refining the use of these instruments according to the protocol and the specific aspects sought. This is in line with current recommendations to prefer the combination of several tools or measurement instruments to report on the skills of young children (Boekaerts and Cascallar, 2006; Perry and Winne, 2006).

## Data availability statement

The raw data supporting the conclusions of this article will be made available by the authors, without undue reservation.

## Ethics statement

The studies involving human participants were reviewed and approved by the Research Ethics Committee of the University of Lille, France (Comité d’Ethique de la Recherche-CER). Written informed consent to participate in this study was provided by the participants’ legal guardian/next of kin.

## Author contributions

ML and JC did the conception and design of the study. ML performed the material preparation and data collection. ML and GG did the data analysis. ML wrote the first draft of the manuscript. JC and CM conducted a more detailed critical review of the work and thanks to their mutual expertise. All authors contributed to the writing of the manuscript, commented on previous versions of the manuscript, and read and approved the final manuscript.

## Funding

This research—and the writing of the manuscript—was funded by support provided to the first author by the Institut National Supérieur du Professorat et de l’Education de l’Académie de Lille Hauts-de-France (INSPE Lille –HdF).

## Conflict of interest

The authors declare that the research was conducted in the absence of any commercial or financial relationships that could be construed as a potential conflict of interest.

## Publisher’s note

All claims expressed in this article are solely those of the authors and do not necessarily represent those of their affiliated organizations, or those of the publisher, the editors and the reviewers. Any product that may be evaluated in this article, or claim that may be made by its manufacturer, is not guaranteed or endorsed by the publisher.

## Footnotes

^1^ https://www.ravensburger.fr/produits/puzzle/puzzle-enfant/puzzles-2x24-p-des-museaux-efficaces-pat-patrouille-09085/index.html

## Appendix A

The support for the realization of the puzzle.

Real size = A4.
